# Part I: Relationship among Training Load Management, Salivary Immunoglobulin A, and Upper Respiratory Tract Infection in Team Sport: A Systematic Review

**DOI:** 10.3390/healthcare9040366

**Published:** 2021-03-24

**Authors:** Markel Rico-González, Filipe Manuel Clemente, Rafael Oliveira, Naia Bustamante-Hernández, José Pino-Ortega

**Affiliations:** 1 Department of Physical Education and Sport, University of the Basque Country (UPV-EHU), 01007 Vitoria-Gasteiz, Spain; 2 BIOVETMED & SPORTSCI Research Group, University of Murcia, 30720 San Javier, Spain; josepinoortega@um.es; 3 Escola Superior Desporto e Lazer, Instituto Politécnico de Viana do Castelo, 4900-347 Viana do Castelo, Portugal; filipe.clemente5@gmail.com; 4 Instituto de Telecomunicações, Delegação da Covilhã, 1049-001 Lisboa, Portugal; 5 Sports Science School of Rio Maior, Polytechnic Institute of Santarém, 2140-413 Rio Maior, Portugal; rafaeloliveira@esdrm.ipsantarem.pt; 6 Research Centre in Sport Sciences, Health Sciences and Human Development, 5001-801 Vila Real, Portugal; 7 Life Quality Research Centre, 2140-413 Rio Maior, Portugal; 8 Department of Dental Medicine, Faculty of Medicine and Dentistry, University of Valencia, 46010 Valencia, Spain; naiabustamante@gmail.com; 9 Faculty of Sports Sciences, University of Murcia, 30720 San Javier, Spain

**Keywords:** immunosuppression, IgA, symptoms, team sports, stress

## Abstract

Immunoglobulin A (IgA), which is the first line of defense against upper respiratory tract viruses, has been related with training load management. This article aimed to systematically identify and summarize (1) the studies that have found a relationship between training load and salivary IgA in team sports, and (2) the studies that have highlighted a relationship between IgA and upper respiratory tract infection (URTI) in team sports. A systematic review of relevant articles was carried out using two electronic databases (PubMed and WoK) until 3 October 2020. From a total of 174 studies initially found, 24 were included in the qualitative synthesis. This systematic review confirmed that lower values of IgA occurred after greater training load (intensity/volume) and congested periods. In this scenario, a low level of IgA was correlated with higher URTI, which makes training load management mandatory to healthcare avoiding immunosuppression. Therefore, physical fitness and conditioning coaches should carefully manage training load progression, avoiding high-intensity sessions in two consecutive days. In addition, they should not program high-intensity training sessions during at least the two days following competition.

## 1. Introduction

Upper respiratory tract infections (URTIs) have been extensively studied in the context of exercise [1], since they are the most prevalent illness reported in athletes [2]. In a theoretical model called the “J” curve, it is hypothesized that moderate exercise is associated with a lower risk of infection, while vigorous exercise performed with frequency or density may lead to an increase in the risk of infection by contributing to a diminishing of immunosurveillance [3]. Some stressors such as high training load, congested match schedules, travels, environmental extremes, or lifestyle are some of the main reasons to explain the exposure to a diminished immunosurveillance [4].

Among others, immunoglobulin A (IgA) is one of the main active antibodies in defending against infectious agents. IgA acts as the first line of defense, inhibiting the bacterial and viral adhesion to epithelial cells and neutralizing bacterial toxins and viruses [5]. In particular, secretory IgA plays a determinant role in mucosal surfaces (e.g., respiratory tract) which is determinant for fighting the entry of many pathogens [6,7]. Despite a great intra- and inter-individual variability of IgA levels in athletes [8], salivary IgA seems to be an effective outcome for controlling the risk of developing URTIs in athletes [4].

Training load and training periodization can be considered some of the stressors responsible for affecting salivary IgA. However, findings in the original studies are inconsistent, and, in some cases, correlations between accumulated training load and IgA are unclear [9,10]. The evidence is also unclear in acute response, since some studies presented no significant changes in salivary IgA concentrations after a match [11,12,13], while others presented a significant reduction in salivary IgA concentrations hours after competition or heavy training [14,15,16].

In the pandemic context, in which SARS-CoV-2 presents high rates of infection by severely affecting the URT, it is determinant to identify how the first line of defense of the respiratory track (i.e., IgA) may vary on the basis of training load imposed in athletes. In fact, team sports players are still training and competing with very congested schedules, leading to accumulated loads that should be considered from an immunological point of view [17]. For that reason, aiming to identify how training load may affect the salivary IgA levels in team sports players, it is important to conduct a systematic review. So far, there has been a narrative review that analyzed the impact of sport related stressors on immunity and illness risk in team sports players [4]. However, as far we may know, no systematic review was conducted about the influence of training load on the IgA levels of team sports players. This may help to understand the consequences of training load on IgA and, ultimately, to provide information or guidelines to protect the players from URTIs.

Accordingly, the aim of this article was to systematically identify and summarize (1) the studies that have found a relationship between training load and salivary IgA in team sports, and (2) the studies that have highlighted a relationship between IgA and upper respiratory tract infection (URTI). We hypothesized that lower values of IgA are present after greater training load periods and congested schedules, making rest strategies necessary after matches and high-intensity training sessions. In addition, we hypothesized that IgA is correlated with URTI, which makes non-congested schedules mandatory.

## 2. Materials and Methods

This systematic review is reported in accordance with the Preferred Reporting Items for Systematic Reviews and Meta Analyses (PRISMA) guidelines [18].

### 2.1. Design

A systematic search was performed by two authors to identify articles published before 3 October 2020 in PubMed and Web of Science. The following search strategy was used (sport, intervention, and population): (“team sport*” OR handball OR hockey OR basketball OR rugby OR soccer OR football OR futsal OR “indoor football”) AND (salivary) AND (immunity OR “Immunoglobulin A” OR sIgA OR “sIgA secretion rate” OR “srIgA” OR “mucosal immunity” OR “upper respiratory symptom*” OR URS). Due to the high number of articles found, the present systematic review summarized all articles performed in team sports (rugby, Australian football, basketball, handball, ice hockey, futsal), while the articles carried out with soccer players were summarized in another systematic review (Part II).

### 2.2. Screening Strategy and Study Selection

After completion of the search, results were compared between researchers (M.R.G. and J.P.O.) to ensure that the same number of articles was found. Then, one of the authors (M.R.G.) downloaded the main data from the articles (title, authors, date, and database) to an Excel spread sheet (Microsoft Excel, Microsoft, Redmond, DC, USA) and removed duplicate records. Subsequently, the same authors screened the remaining records to verify the inclusion/exclusion criteria using a hierarchical approach. The papers were excluded when they met the exclusion criteria in Table 1.

### 2.3. Methodological Assessment

The methodological assessment process was performed by two authors (M.R.G. and N.B.H.) using an adapted version of the STROBE assessment criteria for cross-sectional studies, finding studies eligible for inclusion. Each article was assessed on the basis of 10 specific criteria (see Table 2). Any disagreement was discussed and solved by consensus decision. Each item was evaluated using numerical characterization (1 = completed; 2 = non-completed). As suggested by O´Reilly et al. [19], each study rating was qualitatively interpreted as follows: if score ≥ 7, the study was qualified as having a low risk of bias; otherwise, the study was qualified as having a high risk of bias.

## 3. Results

### 3.1. Identification and Selection of Studies

**Figure 1:**
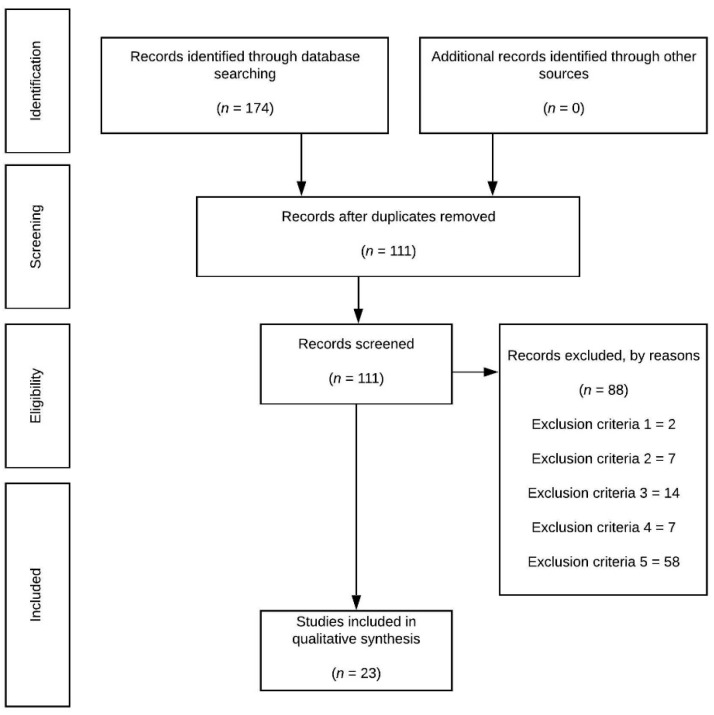
Flowchart.

A total of 174 (PubMed = 67; Web of Sciences = 107) documents were initially retrieved from the databases, of which 63 were duplicated. Thus, a total of 111 articles were downloaded. After screening the titles and abstract against criterion 1 (where applicable), as well as the full text of the remaining papers against the same criterion, two studies were excluded. From the 85 articles, 7, 14, 7, and 58 were ruled out against criteria 2, 3, 4, and 5, respectively. Therefore, 23 studies were included for the qualitative analysis (Figure 1).

### 3.2. Methodological Quality

The quality assessment of the cross-sectional studies can be found in Table 2.

### 3.3. Study Characteristics

Among the selected studies, 17 studies achieved a score higher than 7, meaning a low risk of bias [11,13,21,22,23,24,25,26,27,32,33,34,36,37,38], while seven studies were considered as having a higher risk of bias [10,20,28,30,31,34].

This systematic review included six rugby [10,11,20,28,34,39], two handball [21,24], nine basketball [13,22,26,27,31,33,36,37], three futsal [23,25,32], two hockey [29,38], and two Australian football [30,35] studies.

A detailed description of the 23 studies regarding the sample, the stressor, IgA, and their conclusions is presented in Table 3, Table 4 and Table 5.

Table 3 is organized according to the main stressor of matches. Specifically, when the stressor was matches, they were split according to official matches [11,21,30,32,35], training matches [20,24,30,32], both official and training matches [36].

Table 4 is organized according to the main stressor of training which included training (only general training periods) [10,27,37,38,39], training with strength/resistance [22,23,25,33].

Finally, Table 5 is organized with the last stressor considered which included periods with training and official matches [26,28,29,31]. 

## 4. Discussion

The aim of this systematic review was to investigate the studies that have found (i) a relationship between training load and salivary IgA (sIgA) in team sports, and (ii) a relationship between sIgA and URTI. Among the studies found, the main stressors used in studies were official matches [11,13,21,34,35], training matches [20,24,30,32], simultaneous official and training matches [36], periods of general training [10,27,37,38,39,40], periods of training that included strength training [21,22,24,32], and simultaneous training periods and matches [25,27,28,30]. Although not all studies reported narrow relationship between IgA and training load and between IgA and URTI, a trend was found between training/competition volume/intensity and IgA.

### 4.1. IgA Outcomes

This review found inconsistent results regarding the IgA outcomes. First, it is important to clarify that IgA was analyzed as salivary concentration or secretion. In general, sIgA concentration was measured by enzyme-linked immunosorbent assay (ELISA). The sIgA secretion rate (μg·min^−1^) was calculated by multiplying the absolute sIgA concentration by salivary flow rate (mL·min^−1^), and salivary flow rate was determined by dividing the volume of saliva collected by the duration of the sampling period.

#### 4.1.1. Official Matches

Regarding the studies that analyzed IgA through official matches [11,13,20,21,34,35,41], Lindsay et al. [11] found that three professional rugby matches decreased sIgA concentrations, although not all of them were significant. The same study found that there were decreases over time when analyzing subject by subject, which led to the speculation that some players could possibly be better prepared for matches and, thus, less exposed to immune system fragility. On the one hand, some athletes showed significant levels of muscle damage, stress, and immune system suppression; on the other hand, some athletes did not present such levels. Some additional explanations could be attributed to confidence aspects, experience, or even the lack of match time. Moreover, Coad et al. [35] analyzed 16 Australian Rules Football League matches and found significant decreases in sIgA concentration after 36 h in eight of the 16 matches analyzed (range values = 110.31 ± 72.01–149.35 ± 68.50 μg/mL). In the same way, Mariscal et al. [21] found that one handball official match significantly decreased sIgA concentration (mean difference = 495.5 µg/mL (95%CI = 223.48–767.93)). In contrast, Koch et al. [34] and Moreira, Bacarau et al. [13] found that one rugby and basketball match did not present significant decreases, respectively. Some possible differences in the results could be associated with the specificities of the sport, such as technical/tactical actions, duration of the matches, intensity of the matches, and some contextual factors, such as the quality of the opponents, match location (home versus away), and match result (weather the team is winning, loosing, or drawing) that could influence the results. In addition, sIgA following acute exercise did not change as previously reported [42], but intensity of the exercise can also be a major factor affecting sIgA outcomes. Furthermore, the volume and composition of fluid consumed during the match or training and sweat rates could influence the results. Lastly, the timing of saliva sample collection may also have been a factor in the outcomes. If the collection is immediately before the training or match, it may not reflect a true resting value because players could be in a higher state of excitement, which can arouse psychological activity [42]. The same could be applied after the match. For instance, only one study did not collect immediately after the match, but 36 h after, and still found significant decreases in sIgA concentrations (pre- to post-match, 110.31 ± 72.01–136.74 ± 63.16 μg/mL) [34] while the other studies collected saliva samples 5–15 min after the end of the match [11,13,20,33].

#### 4.1.2. Training Matches

When the stressor was training or friendly matches, Lindsay et al. [20] (sIgA concentration pre- to post-match 409 ± 223 to 414 ± 255 μg/mL) and Caetano Júnior et al. [24] (pre- to post-match values not described) found no significant differences after one rugby and handball match, respectively, while Arruda et al. [32] found significant decreases after one futsal match (pre- to post-match sIgA concentration, 715.6 ± 214.8–463.9 ± 154.6 µg/mL) and Coad et al. [30] found significant decreases after 12 and 36 h (sIgA concentrations pre-match = 290.21 ± 165.28; post 12 h = 147.20 ± 83.94; post 36 h = 113.32 ± 95.26 µg/mL) of three different Australian football matches. On one hand, the physical demands of the rugby and handball matches also included periods of recovery between efforts and relatively short durations of action, which, associated with the characteristics of friendly matches, could have influenced the outcomes in mucosal parameters; however, this was not investigated properly in both studies. As mentioned before, other justifications could be the psychological factor, once the matches were in training or simulated conditions, and the exercise intensity/duration [43]. On the other hand, a futsal training match, due to higher intensity, may be sufficient to decrease IgA. Future studies are required to better understand these findings.

#### 4.1.3. Official and Training Matches

In addition, one study compared official and training matches [36]. The authors found that no significant changes in sIgA concentrations were observed across either the simulated (pre- to post-match, 494 ± 99–635 ± 137 μg/mL) or the official matches (pre- to post-match, 457 ± 68 552 ± 59 μg/mL). Beyond the explanations given before in an attempt to justify the findings, it is suggested that the glucocorticoid family of steroids, such as cortisol, could be responsible for controlling IgA changes [44]; however, both studies failed to prove that cortisol does play such a role in controlling IgA acute responses of exercise. Instead, they showed an inverse relationship between salivary IgA and cortisol concentrations in young adults without exercise [45]. 

#### 4.1.4. General Training and Training with Strength/Resistance

Regarding the studies that only analyzed periods of general training [10,27,37,38,39,40], Moreira et al. [37] found significant decreases in sIgA concentrations after a 17 day period, which was performed with maximal intensity (pre- to post-training, 541 ± 226–381 ± 111 μg/mL), but no training program was revealed. Orysiak et al. [38] also analyzed a 17 day period. During that period, three saliva collections were taken (beginning, day 9, and day 13). Higher load was imposed between the first two assessments when compared with the period between the last two assessments. As expected, it was found that the first period significantly decreased IgA (116.17 ± 51.66–113.99 ± 76.57 μg/mL), especially when compared with the last (122.84 ± 82.87 μg/mL). In contrast, Nunes, Crewther, Viveiros et al. [33] found that a 50 day period of resistance training with progressive load through the weeks decreased IgA concentrations (values not described), and Milanez et al. [23] found that 5 weeks with strength training, physical training, and technical/tactical training also decreased sIgA concentrations (baseline = 52.2 ± 32.1, week 1 = 55.5 ± 32.3, week 2 = 78.2 ± 30.3, week 3 = 38.8 ± 16.6, week 4 = 69.3 ± 34.9, and week 5 = 59.8 ± 31.4 μg/mL) and secretions (baseline = 52.2 ± 59.4, week 1 = 49.0 ± 27.1, week 2 = 61.0 ± 21.9, week 3 = 30.3 ± 13.7, week 4 = 56.3 ± 35.3, week 5 = 49.0 ± 27.3 μg/mL). On the other hand, other studies that included strength training found no significant differences [22,25]. Moreira et al. [25] only collected sIgA once a week, which affected the results, and Nunes et al. [22] did not increase the load over time unlike the study of Milanez et al. [23], which found some significant results.

#### 4.1.5. Training and Official Matches

Some studies analyzed in-season periods that included training and matches [25,30]. He et al. [31] found that 4 weeks of intensive training period and 2 weeks with matches decreased sIgA when compared to recovery week concentrations (pre to post four weeks of training = 146.7 ± 18.0–144.9 ± 22.7; match 1 = 142.9 ± 11.9; match 2 = 153.2 ± 18.0; recovery weeks range values = 204.3 ± 20.5–210.7 ± 15.0 ug/mL). Another study found decreases in sIgA secretion (106 ± 20–92 ± 21 ng·mL^−1^), while absolute sIgA concentration increased (587 ± 94–720 ± 153 μg/mL) after 4 weeks of training with one match per week [26]. In this case, the increase in sIgA concentration could be associated with the lower load applied in the last week of training, which could have influenced results. If the authors collected saliva samples 1 week before, they probably would have found decreases in sIgA concentration [26].

### 4.2. Relationship between Salivary IgA and URTI

Few studies analyzed in the present systematic review found relationships between IgA and URTI [10,25,41]. However, these findings are inconsistent because other studies [28,29,37,38] found no statistically significant correlations. Furthermore, in general, the studies revealed methodological limitations in analyzing the relationship such as different times of salivary collection and different periods of analysis.

Moreover, it is important to reinforce that IgA is an antibody and an immune marker found on the mucosal surface, including saliva [46]. For that reason, IgA works as a protector against viruses and antigens [47]. Therefore, low levels of sIgA could be one of the reasons causing URTIs [10,25,41].

The study of Moreira, Moura et al. [25] analyzed a 4 week period of intensive training. Those authors hypothesized that increments in training load might decrease sIgA levels and increase symptoms associated with URTI, and they confirmed this hypothesis, finding such a correlation in week 4. This study indicated that some athletes could have decreased sIgA and, consequently, be exposed to higher risk of URTI. This argument was also stated by another study, which found that a low sIgA secretion rate may be considered a risk factor for the development of URTI [48]. In addition, it was found that higher load and intensity could also contribute to URTI occurrence [41,48]. The Tiernan et al. [10] study also corroborated this relationship, finding that a decrease ≥65% in sIgA contributed to a higher risk of developing URTI in the following 2 weeks.

In addition, Cunniffe et al. [28] did not present significant correlations, but the authors found lower sIgA concentrations in players who reported higher incidences of URTI than players who were URTI symptom-free.

Meanwhile, other studies could not present such results, which does not mean that a biological relationship does not exist [49]. Moreira et al. [37] analyzed a 17 day training period with respect to sIgA levels in basketball players, and only one episode of URTI symptoms was reported with no association with sIgA levels. Orysiak et al. [38] also analyzed the effects of a 17 day training period on sIgA levels in ice hockey players and found no statistically significant correlations between the URTI incidence and sIgA. The same study justified this by the variation in mucosal markers between players, which could have affected the results, and the authors reinforced an individual analysis, player by player. In addition, the small sample size may have also affected the results. Another study also confirmed these findings, in which lower concentrations of sIgA were observed in the athletes with URTI symptoms compared to the healthy state [29]. This helps to explain the previous suggestion to monitor mucosal markers at an individual level [11,13,29].

From the aforementioned studies, the hypothesis of the present study that IgA correlated with URTI is supported. It was possible to confirm that low levels of sIgA could be associated with a higher risk of URTI. In other words, it is recommended to avoid long periods with low levels of sIgA and, consequently, higher risk of URTI.

### 4.3. Relationship between Salivary IgA and Training Load

Another main finding of the present systematic review was that a higher training load was associated with low levels of salivary IgA, which confirmed our hypothesis. As suggested by Mariscal et al. [21], the determination of IgA concentrations could help to identify a higher or excessive training workload and could also determine the risk of URTI in professional athletes.

One of the aims of the Tiernan et al. [10] study was to investigate the relationship between sIgA and training load, and they hypothesized that there would be an inverse relationship between sIgA and training load. However, while there were no significant associations found between these markers, the same study showed that, 1–2 weeks before the decrease in sIgA, the training load increased by 49%. One justification for the lack of association between sIgA and training load could be associated with appropriate training load management, avoiding low levels of sIgA by ensuring sufficient recovery [50]. Furthermore, Lindsay et al. [20] found a relationship between sIgA secretion rate and player load. No explanations for this result were given by those authors. In addition, Mariscal et al. [21] did not find any relationship, but they found that participation for more than 30 min showed a statistically significant decrease in IgA. The authors suggested that cumulative activity could influence some biomarkers such as IgA; therefore, rotation of players should be considered for team sports to avoid this negative outcome.

The nonsignificant findings of previous studies [10,20,21] contradicted others [2,20,25,41]. For instance, Moreira, Moura et al. [25] found a significant association between training load and URTI in futsal athletes. As training load decreased, the URTI symptom severity decreased, suggesting that higher training periods led to athletes becoming more susceptible to developing an URTI. The previous statement was supported by other studies finding that higher load and intensity could also contribute to URTI occurrence [41,48]. The previous findings lead us to reinforce that higher levels of URTI are associated with low levels of salivary IgA, which are also associated with higher levels of training load.

Further information was given by Coad et al. [35], who revealed that lower values of sIgA occurred with higher player load values in rugby players. Moreira, Mortatti et al. [51] demonstrated that, compared with a post-season detraining phase, sIgA decreased when athletes were involved in preseason training and in-season training and competition. It is important to reinforce that this study analyzed a congested period (more than one match per week), which reinforces one of our study hypotheses. For instance, Morgans et al. [52] also revealed that compromised immunological function appeared during a highly congested soccer season. Despite soccer studies not being analyzed in the present systematic review, this study is mentioned because it supports the previous finding, necessitating more research in other sports. A less recent study found that 6 weeks of preseason training and a 10 week in-season schedule for American college football athletes significantly decreased sIgA and increased the incidence of URTI [53].

### 4.4. Study Limits and Future Directions

As mentioned by Nunes, Crewther, Ugrinowitsch et al. [33], there are some confounding effects of other variables such as type of training, periodization, and psychological factors that can influence results and should be considered for future research. However, except for He et al. [31], Koch et al. [34], and Yamauchi et al. [39], who used university or college athletes, and Moreira et al. [37], who included professional athletes and staff members, all studies analyzed in the systematic review represented the actual training environment of athletes and the inherent limitations.

In addition, there are other factors that can contribute to decreases in sIgA and, thus, increase the risk of infection, such as exposure to pathogens, mucosal damage, and environmental conditions (i.e., air pollutants and pollens). These factors could be involved in the higher incidence of respiratory infections [37].

However, the 24 studies included in this systematic review did not analyze studies on soccer due to the large number of published scientific manuscripts; therefore, all conclusions should be carefully interpreted by soccer researchers, coaches, or their staff members.

The present research only analyzed one study [28] that included a full season; thus, future studies should analyze full seasons in different sports.

Furthermore, the small sample size was a limitation identified in the studies included in the present systematic review, necessitating more research with a larger number of participants.

Moreover, future studies are required to clarify the adaptations in sIgA produced by different training protocols and types, as the present research could not highlight this information due to few studies clearly presenting the training protocol. Furthermore, more insight is required regarding congested periods (i.e., weeks with two or more matches) in order to clarify, expand, and confirm the present results, since no study in team sports other than soccer analyzed this variable.

The main confirmation of this study is that sIgA should be used as a measure to analyze the risk of a subsequent URTI in different sport teams. With that information, coaches, staff, and the scientific community could imply appropriate prescription and management of training load through a proper periodization. Thus, training and performance can be optimized, and players can be better prepared for competition. Saliva sample collection is also noninvasive, easy to collect, and time-efficient; thus, it could be easily implemented for training quantification [46]. Lastly, as suggested by Tiernan et al. [10], sIgA should be collected before the start of the season as a reference baseline for future collections and better interpretation of outcomes. Then, sIgA should preferably be collected before and after matches or in the beginning and ending of a microcycle. The results of sIgA and its relationships with URTI and training load should be individually analyzed for better interpretation.

## 5. Conclusions

The present systematic review confirmed both hypotheses, i.e., that lower values of IgA occurred after greater training load periods, and that IgA was correlated with URTI. In brief, IgA decrement is related with both greater intensity/volume and congested schedules. Therefore, physical fitness and conditioning coaches should carefully manage training load progression, avoiding high-intensity sessions on two consecutive days or following matches. In addition, since competition may suppose an additional stressor that may affect IgA, no high-intensity training sessions should be performed during at least the two days following competition.

### Strength of the Study

The number of studies aimed at extracting hormone levels and/or antibodies (e.g., IgA) seems to be growing in popularity [4]. Antibodies are proteins that the immune system makes to fight attacks by bacteria, viruses, and toxins. The mucosal surfaces are protected by a network of organized structures located in the gut, urogenital tract, oral cavity, and respiratory system, collectively known as the mucosal immune system [14].

The production of secretory IgA has been considered as the “first line of defense” of the mucosal immune system against pathogens [54]. However, since different articles have highlighted a relationship between training/match load and IgA levels, and between lower levels of IgA and URTI, physical fitness and conditioning coaches should ensure an optimal training load management to avoid immunosuppression.

On this note, a recently published systematic review about recommendations and best practices to return to competition after COVID-19 lockdown briefly highlighted the importance of training load management to avoid effects on IgA [17]. However, to the authors’ knowledge, despite the large number of articles published aimed at assessing the influence of training and competition load on sIgA, there is a lack of systematic reviews that summarize key findings allowing decision-making. Therefore, the present systematic review can allow physical fitness and conditioning coaches of different team sports (rugby, Australian football, basketball, handball, futsal, and hockey) to quickly get information on how to manage load in order to avoid immunosuppression.

## Figures and Tables

**Table 1:** Inclusion/exclusion criteria.

No.	Inclusion Criteria	Exclusion Criteria
1	Samples were taken from salivary test	Samples collected from non-salivary test (e.g., blood samples)
2	Biomarkers related to immunology (IgA)	Biomarkers related to hormones (testosterone, cortisol) or immunology (not IgA)
3	Articles in which the relationship between training load and IgA was assessed without other factors	Articles that assessed the influence of another factor (e.g., nutritional intervention, recovery strategies) in IgA response or the effects of IgA in other contexts (e.g., oral health)
4	Original articles	Nonoriginal research papers (i.e., systematic reviews, conferences, or meetings)
5	Articles developed with team sport athletes (rugby, basketball, futsal, Australian football, ice hockey, or handball) more than 18 years old.	Articles developed with soccer players or with non-team sport athletes

**Table 2:** Methodological assessment of the included studies.

Reference	1	2	3	4	5	6	7	8	9	10	Quality
Lindsay et al. [20]	1	0	1	1	1	1	1	0	0	1	Low
Lindsay et al. [11]	1	0	1	1	1	1	1	1	1	0	High
Mariscal et al. [21]	1	1	1	1	1	1	0	1	1	1	High
Nunes et al. [22]	1	1	1	1	1	1	1	1	1	1	High
Milanez et al. [23]	1	1	1	1	1	1	1	1	0	0	High
Caetano Júnior et al. [24]	1	0	1	1	1	1	1	1	0	1	High
Moreira et al. [25]	1	1	1	1	1	1	1	1	0	1	High
Moreira et al. [26]	1	1	1	1	1	1	1	1	1	1	High
Nunes et al. [27]	1	1	0	1	1	1	1	1	0	1	High
Cunniffe et al. [28]	1	0	0	1	1	1	1	1	1	0	Low
Orysiak et al. [29]	1	1	1	1	1	1	1	0	0	1	High
Coad et al. [30]	1	0	1	1	1	1	1	0	0	1	Low
He et al. [31]	1	0	1	1	1	1	1	0	0	1	Low
Arruda et al. [32]	1	1	0	1	1	1	1	1	0	1	High
Nunes et al. [33]	1	1	1	1	1	1	1	0	0	1	High
Tiernan et al. [10]	1	0	1	1	1	1	1	1	0	0	Low
Moreira et al. [13]	1	1	1	1	1	1	1	0	1	1	High
Koch et al. [34]	1	1	0	1	1	1	1	0	0	0	Low
Coad et al. [35]	1	0	0	1	1	1	1	0	0	1	High
Moreira et al. [36]	1	1	1	1	1	1	1	0	0	1	High
Moreira et al. [37]	1	1	1	1	1	1	1	1	0	0	High
Orysiak et al. [38]	1	1	1	1	1	1	1	1	1	1	High
Yamauchi et al. [39]	1	1	0	1	1	1	1	1	1	1	High

**Note:** Provide in the abstract an informative and balanced summary of what was done and what was found (item 1). State specific objectives, including any prespecified hypotheses (item 2). Give the eligibility criteria, and the sources and methods of selection of participants (item 3). For each variable of interest, give sources of data and details of methods of assessment (measurement). Describe comparability of assessment methods if there is more than one group (item 4). Explain how quantitative variables were handled in the analyses. If applicable, describe which groupings were chosen and why (item 5). Give characteristics of study participants (item 6). Summarize key results with reference to study objectives (item 7). Discuss limitations of the study, considering sources of potential bias or imprecision. Discuss both direction and magnitude of any potential bias (item 8). Give a cautious overall interpretation of results considering objectives, limitations, multiplicity of analyses, results from similar studies, and other relevant evidence (item 9). Give the source of funding and the role of the funders for the present study and, if applicable, for the original study on which the present article is based (item 10). Score: 1 = Yes; 2 = No.

**Table 3:** Immunoglobulin A responses after match load and its relationship with upper respiratory tract infection (URTI).

Ref.	Sample	Stressor			Immunoglobulin (IgA)			Study Limits	Lessons Learned and Concluding Remarks
		Test	Volume	Intensity	Salivary Test	Outcomes	IgA–URTI Relationship		
**Official Matches**									
Rugby									
Lindsay et al. [11]	24 professional rugby players from New Zealand provincial team.	3 professional rugby games.	Game 1 = 72.1 ± 15.4; Game 2 = 66.6 ± 17.0; Game 3 = 74.4 ± 18.4 min.	Game time, total distance covered (m), player load (au), and number of impacts were as follows: Game 1 = 5191, 544.7, 25; Game 2 = 5368, 565.2, 22; Game 3 = 5346, 585.2, 26.	Saliva samples were collected ~120 min pre-game and ~30–40 min post-game.	Post-game decrements (*p* < 0.001). sIgA decreases for game 2 (*p* = 0.019). Mean sIgA decreases following all games.	No.	Small sample size and only 3 games were analyzed.	Significant decreases in sIgA concentration and secretion were observed for game 2. Post-game secretion rate is affected by pre-game rate and number of impacts.
Koch et al. [34]	16 men’s college rugby players.	1 regulation rugby game.	80 min.	Intensity of the match was not controlled.	Saliva samples were collected ~10 min before pre-match warm-up and within 5 min post-game.	No significant pre-post-game changes in sIgA were observed (−13%).	No.	No load quantification by athlete and fluid consumption during the game.	Strenuous physical activity, such as a competitive rugby game, does not affect sIgA.
Australian Football									
Coad et al. [35]	18 elite male Australian Rules Football League athletes.	16 consecutive matches.	1 match per week (4 × 30 min quarters).	Player load range values from 1392.14 ± 139.46 to 1263.89 ± 180.69 AU.	sIgA was measured at 36 h post-match before daily activities.	Significant (*p* < 0.05) effects compared with pre-season baseline sIgA. After matches, a substantial suppression of sIgA was found compared with pre-season baseline.	No.	Small sample size. Only 36 h post-match periods were monitored, which can influence the results interpretation.	Matches may delay sIgA recovery beyond 36 h post-match for full recovery and may be at higher risk of illness during the initial 36 h post-match.
Basketball									
Moreira et al. [13]	20 under-19 male basketball players competing in the State Basketball Championship (São Paulo, Brazil).	1 basketball match, plus 30-min warm-up.	Each match included 4 × 10 min quarters with an interval of 2 min between quarter, plus a 30 min warm-up.	Intensity of the match was not controlled.	Saliva samples were collected before the warm-up and approximately 10–15 min after the end of the match.	sIgA did not change in response to the match.	No.	Small sample size, and changes in the hydration status and their possible influence on saliva flow rate were not controlled.	A basketball match does not compromise sIgA.
Mariscal et al. [21]	21 elite female handball players in the Spanish national league divided into two groups: wingers/forwards (n = 10), and defenders (n = 11).	1 official match.	Mean playing time was 25.2 min which was 42% of the match’s duration (60 min with 15 min rest between halves).	Intensity was not controlled.	Saliva samples were collected 5 min before and 10 min after the last official match	IgA fell (*p* < 0.01) at the end of the match. Defenders exhibited the lowest IgA concentrations after the match. Larger changes in IgA were seen in those who played for more than 30 min.	No.	Small sample size and only 1 game was analyzed. Moreover, the phase of the menstrual cycle was not controlled.	Competitive handball matches increase physiologic stress in females, resulting in decreased immunocompetence.
**Training matches**									
Rugby									
Lindsay et al. [20]	11 rugby players from 2 senior men’s division in New Zealand.	Elite amateur rugby game that may be used to manage player recovery.	80 min	Impacts = 46 ± 25. Distance = 6029 ± 690 (47% at <8 km/h, 27% at 8–13 km/h, 17% at 13–18 km/h, 6% at 18–23 km/h, and 2% at >23 km/h).	Saliva samples were collected 24 h before the game, immediately post-game, and at 17, 25, 38, 62, and 86 h post-game.	No significant differences in sIgA concentration before and after the game (409 ± 223 to 414 ± 255 μg/mL).	No.	Small sample size, and only one game was analyzed.	Low-intensity matches led to lack of change in sIgA.
Australian Football									
Coad et al. [30]	11 elite Australian Rules Football League athletes.	3 matches during the pre-season.	Each match, 4 × 30 min quarters.	Player load during match 3 (1266 ± 124.6 AU) was significantly (*p* < 0.01) greater than in match 1 (1096 ± 115.1 AU) and match 2 (1082 ± 90.4 AU).	Saliva samples were collected across each match 24 h and 1 h pre-match and 1, 12, 36, and 60 h post-match.	Across match 3, sIgA was significantly (*p* < 0.01) suppressed at 2 post-match measures (12 and 36 h) compared with pre-match measures (24 and 1 h), which coincided with significantly (*p* < 0.01) elevated player load.	No.	Small sample size, and only 3 matches were analyzed from pre-season.	The findings indicated that an increase in player load during the match resulted in compromised post-match mucosal immunological function.
Handball									
Caetano Júnior et al. [24]	14 male handball players.	1 simulated handball match.	20 min of warm-up, 1 match composed of two halves of 30 min with 5 min interval for recovering.	Players indicated that match intensity was difficult or very difficult according to the rated perceived exertion scale.	Saliva samples were collected ~10 min before (~4:00 p.m.), ~5 min after the match, and after 2 h of recovery.	The saliva samples collected before and after match showed no significant difference.	No.	Small sample size, and only 1 match was analyzed.	No significant spectral differences to saliva sample collected pre- and post-match, as well as after 2 h of recovery.
Futsal									
Arruda et al. [32]	10 elite futsal players from a Brazilian under-20 team.	1 simulated futsal match.	80 min.	Session rated perceived exertion revealed 400.00 ± 37.71 AU of training load.	At rest, before the start of the match and 5–10 min after the match.	sIgA before = 715.6 ± 214.8; sIgA after = 463.9 ± 154.6 μg/mL (*p* < 0.05).	No	Small sample size, and only 1 match was analyzed.	Simulated futsal matches induce a high level of stress, moderated by the high-magnitude internal load and by decreasing the level of SIgA.
**Studies with both official and training matches**									
Basketball									
Moreira et al. [36]	10 elite young male basketball under-19 players playing in the main São Paulo (Brazil) competition.	15 weeks with 3 simulated training matches and 2 official matches.	5 days, 2 trainings/day, 90–120 min/training 1 match (4 × 10 min quarters, with 2 min rest and 30 min warm up).	Match intensity was controlled through session rated perceived exertion and revealed that official matches were significantly (*p* < 0.05) greater than the simulated training match.	30 min before the pre-match warm-up and 10 min after each match.	No significant changes in sIgA concentrations were observed across either the simulated or official matches.	No.	Small sample size.	No changes or differences in sIgA were noted between pre- and post-moments across all matches.

**Note:** AU = arbitrary units; sIgA = salivary immunoglobulin A.

**Table 4:** Immunoglobulin A responses after training load and its relationship with URTI.

Ref.	Sample	Stressor			Immunoglobulin (IgA)			Study Limits	Lessons Learned and Concluding Remarks
		Test	Volume	Intensity	Salivary Test	Outcomes	IgA–URTI Relationship		
**Training**									
Rugby									
Tiernan et al. [10]	19 male elite rugby union players.	10 week training.	Players were trained 4–5 days a week, with multiple training sessions a day.	Approximately 1800–2600 AU weekly rated perceived exertion.	Saliva samples were collected twice a week, Monday and Friday, within 1 h of the players waking up. before training.	No significant differences in weekly sIgA levels were found over the 10 week period.	The likelihood of suffering from an URTI increased when sIgA significantly decreased (*p* = 0.046).	The data collection was only related to the pre-season. Moreover, the sample size was small.	A decrease in >65% of sIgA meant players were at risk within the following 2 weeks of contracting an URTI.
Yamauchi et al. [39]	32 male collegiate rugby football players.	1 month training camp.	2 training sessions (morning and afternoon sessions) each day and lasted approximately 2 h.	Intensity was not controlled.	All saliva samples were collected every day before breakfast at 6:30 a.m.	sIgA secretion rate was significantly decreased from baseline 1 day before and 1, 2, 3 days after the day of first Epstein–Barr virus expression during the camp (*p* < 0.05).	No.	The lack of control of medications and supplementations, along with the small sample size.	The number of URTIs increased along with the Epstein–Barr virus expression and the decrease in sIgA, suggesting that the appearance of symptoms is associated with reactivation of Epstein–Barr virus and reduction in sIgA during training.
Basketball									
Nunes et al. [27]	19 elite female basketball players on the Brazilian National Team.	12 weeks.	Volume and intensity progression along the periodization plan.	Weeks 1–3 = moderate. Weeks 4–6 = moderate to high intensity. Weeks 8–12 = speed agility.	1 day before and 1 day after 12 week intervention.	Salivary measures did not change despite the fluctuations in the training program.	No.	The frequency of saliva sampling could influence the results obtained.	The periodized training plan promoted improvement in physical performance avoiding major variations on IgA.
Moreira et al. [37]	10 athletes and staff members from a national basketball team.	17 day training period.	Athletes completed a mean training volume of ~25–30 h per week.	Coaches assumed that the athletes performed all sessions with maximal intensity, and, in this case, the training volume would represent the actual training intensity.	Saliva samples were collected at the beginning of the preparation for the Pan American Games, and 1 day before the first game, at rest, in the afternoon (3:30 p.m.).	Decrease in sIgA levels following training was greater in athletes than in support staff; however, no significant differences between the 2 groups were detected. Significant decreases were found after the period analyzed (*p* < 0.05).	No.	No training program exercises were revealed. There was an absence of observations in sIgA levels on motor performance tests. The sample size was small.	A situation of combined stress for an important championship was found to decrease the level of sIgA-mediated immune protection at the mucosal surface in team members, with greater changes observed in the athletes.
Ice Hockey									
Orysiak et al. [38]	12 young Polish ice hockey national team members.	17 day training period with 2 phases: (1) intensification phase: (days 1–8); (2) tapering (days 9–17).	Both phases of training were ~41.5 h: 13.5 h of strength training and 28 h of training on ice. On days 14 and 15, they played (full-time) friendly international matches.	Session rated of perceived exertion range values: 1260 ± 721 AU and 990 ± 719 AU in phase 1 and phase 2, respectively.	Before training camp. 9 days after the training intensification and on day 13. Saliva collection took place after a minimum of 12 h after the last training.	A significant increase from sIgA1 to sIgA2 in the third collection compared with the second (114.45 ± 33.00 vs. 77.49 ± 27.29 and 88.97 ± 25.33 vs. 71.65 ± 32.44 U, respectively).	There were no statistically significant correlations between the URTI incidence and sIgA.	Food and alimentary habits were not controlled, along with the small sample size.	The tapering period positively affects the mucosal immune function, especially sIgA1 and sIgA2 concentrations, with no significant change in frequency of URTI in young ice hockey players.
**Training with strength/resistance**									
Basketball									
Nunes et al. [22]	14 female basketball players Brazilian National Team.	3 different resistance exercise protocols.	Participants attended 4 assessments over a period of 40 day.	Endurance (4 × 12, 60% of 1RM, 1 min rest) Hypertrophy (1 set of 5RM, 1 set of 4RM, 1 set of 3RM, 1 set of 2RM, and 1 set of 1RM. 3 min rest followed by 3 sets of 10RM with 2 min rest period). Power (3 sets of 10 reps, 50% 1RM, 3 min rest).	sIgA samples were collected at 7:30 a.m., pre-exercise at 9:30 a.m., postexercise, and at 5:30 p.m.	No differences in sIgA concentrations were observed across the 3 schemes at any time (*p* > 0.05).	No.	Menstrual cycle not controlled, small sample size, and no randomized order in schemes.	There were no changes in sIgA concentrations for any workout scheme.
Nunes et al. [33]	12 elite female basketball players.	50 day period of resistance training.	The volume of the 50 day period of resistance training was not described.	Endurance: 3 weeks (3/4 × 15/20). Strength: 2 weeks (3/6 × 1/10). Power: 2 weeks (3/6 × 6/10, 30–50% 1RM).	Saliva samples were collected at 7:00 a.m., 9:30 a.m., 11:00 a.m., and 5:30 p.m. both pre- and post-training, after 2 full days of complete rest.	IgA concentrations were lowered at 9:30 and 11:00 a.m. (*p* < 0.05).	No.	Small sample size, and participants were subjected to other conditioning practices.	A periodized approach to resistance training increased muscle performance in elite female basketball players and only minor changes in the salivary IgA were noted.
Futsal									
Milanez et al. [23]	13 top-level professional female futsal players who were second place in the Brazilian National League in 2009.	5 training weeks in pre-season period.	Resistance: 4 times/week (30–40 min/day). Together, 5 technical/tactic sessions/week (80–120 min). Week 4: resistance decrease to 2 times. Last 2 weeks: volume maintenance.	Resistance (3 × 15 at 70%, rest 45 s. Progressive load in the 3 first weeks (~360–425 AU) while weeks 4 and 5 had a load decrease to ~300 and 250 AU for rated perceived exertion, respectively.	Saliva samples were collected 1 week before the start and prior the last training session of each week at the same time of the day.	There was a significant increase in training load, monotony, and training strain in week 3, with a concomitant and significant reduction in percentage variation of sIgA concentration and secretion rate (*p* < 0.05).	No.	Small sample size.	A link among training load, sIgA levels, and stress symptoms was evident in a nonlinear fashion. Above ~435 and ~3160 AU per session rated perceived exertion and strain, there was a decrease in SIgA levels.
Moreira et al. [25]	12 elite Brazilian futsal players.	4 weeks of intensive training during the competitive season with 27 training sessions performed.	Week 1 = 8 sessions (1095 min). Week 2 = 9 sessions (12,967 min). Week 3 = 6 sessions (755 min). Week 4 = 4 sessions (555 min).	First 2 weeks, higher intensity. Weeks 3–4, load (frequency/volume) was reduced.	Before each week start and at least 36 h after the last training).	No significant differences were observed for sIgA during the study (*p* > 0.05).	The relative change in sIgA absolute was associated with the URTI severity during week 4 (r = −0.74; *p* < 0.05).	The collection of samples after fasting overnight was not applied along with the small sample size.	Futsal athletes were more susceptible to high URTI symptom severity in the periods of higher training intensity and volume. A reduction in training load before competitions is an appropriate strategy to minimize URTI symptoms, ensuring the athlete’s ability to train and compete.

**Table 5:** Immunoglobulin A responses during both training and match load and its relationship with URTI.

Ref.	Sample	Stressor			Immunoglobulin (IgA)			Study Limits	Lessons Learned and Concluding Remarks
		Test	Volume	Intensity	Salivary Test	Outcomes	IgA–URTI Relationship		
Rugby									
Cunniffe et al. [28]	31 elite rugby union players.	48 weeks (full season).	34 games and 345 h of scheduled training.	Monthly comparisons during the season. The peak values were reported for February (10,914 ± 831 AU) before decreasing again until the close of the season (May = 5177 ± 369 AU).	Saliva IgA (n = 11) was taken at regular monthly intervals across the season.	Decreases in sIgA were observed during August (20%), December (29%), and February (9%).	Despite lower mean sIgA concentration in players who reported incidences of URTI, no significant correlation was found between absolute sIgA and URTI incidence.	The nonuse of a control group.	Higher stress-induced contributed to reductions in mucosal immunity, which, when lowered, predisposed rugby players to increased risk of illness.
Basketball									
Moreira et al. [26]	15 basketball players.	4 weeks during the competitive season with 1 game per week.	90–120 min per session, 2 sessions per day, and 5 days per week.	Weeks 1–4: 656 ± 92, 680± 83, 548 ± 37, and 479 ± 34 AU of rated perceived exertion, respectively.	Saliva samples were collected at rest, in the afternoon, at the same time of day both before and after the investigated period.	Decreases in sIgA secretion reduced, while absolute sIgA concentration increased after four weeks (587 ± 94 to 720 ± 153 and 106 ± 20 to 92 ± 21 μg/mL, respectively).	No.	The inability to monitor the salivary parameters each week. Correlations over the 4 time points were not applied, and the sample size was small.	Initial levels of sIgA secretion were negatively and significantly correlated with the signs and symptoms of stress at week 4, which could indicate that the athletes with low levels of sIgA secretion were more susceptible to be affected by different stressors.
He et al. [31]	8 members of National Taichung University basketball team in Taiwan.	11 weeks were analyzed (4 weeks of training, 3 weeks of competition, and 4 weeks of recovery with no training sessions).	Weeks 1–4: duration was ~540 min per week; weeks 5–7: duration was 300 min per week.	Weeks 1–4: higher-intensity training sessions. Weeks 5–7: intensity reduced where 2 matches were played but no metric was used to control intensity.	Saliva samples were collected at rest and before the start of practice or competition, in the afternoon, at 7 time points during the intense training, competition and recovery period.	Significant decreases in sIgA secretion rates and absolute concentrations were observed during the training and competition period.	No.	Small sample size.	Intensive training and competition have adverse effects on the mucosal immunity in basketball players.
Ice Hockey									
Orysiak et al. [29]	27 under-17 ice hockey players from the Athletic High School of the Polish Ice Hockey Federation	24 weeks from competitive season.	In competitive phases, the hockey players participated in 5 weekly training sessions (120–180 min). They also played 1 or 2 matches per week.	Highest training load was found at the beginning of the period (from sampling 1 to 2), and then lower until sampling4. High training loads were observed between sampling 5 and 6, as well as between 7 and 8.	8 times every 3–4 weeks for 21 weeks (before start of the season, weeks 4, 6, 8, 11, 14, 18, 21). A minimum of 12 h after the last training.	Athletes had a lower sIgA concentration at the time when they had symptoms of URTI.	No.	There were some congested periods which were not analyzed, specifically. Moreover, the sample size was small.	Lower concentrations of sIgA during URTI in athletes were observed, which may confirm the suppression of mucosal immunity and initiation responses to pathogenic infections by innate immunity.

## References

[1] Ahmadi S., Brietzke C., Silveira R. (2018). Aspects of Physical Training Related with Upper Respiratory Tract Infections: A Review. Man. Ther. Posturology Rehabil. J..

[2] Gleeson M. (2007). Immune Function in Sport and Exercise. Immune Funct. Sport Exerc..

[3] Nieman D.C. (1997). Risk of Upper Respiratory Tract Infection in Athletes: An Epidemiologic and Immunologic Perspective. J. Athl. Train.

[4] Keaney L.C., Kilding A.E., Merien F., Dulson D.K. (2018). The Impact of Sport Related Stressors on Immunity and Illness Risk in Team-Sport Athletes. J. Sci. Med. Sport.

[5] Lamm M.E. (1997). Interaction of antigens and antibodies at mucosal surfaces. Annu. Rev. Microbiol..

[6] Lamm M.E., Nedrud J.G., Kaetzel C.S., Mazanec M.B. (1995). IgA and Mucosal Defense. APMIS.

[7] Underdown B.J. (1986). Immunoglobulin A: Strategic Defense Initiative at the Mucosal Surface. APMIS.

[8] Neville V., Gleeson M., Folland J.P. (2008). Salivary IgA as a Risk Factor for Upper Respiratory Infections in Elite Professional Athletes. Med. Sci. Sports Exerc..

[9] Blume K., Körber N., Hoffmann D., Wolfarth B. (2018). Training Load, Immune Status, and Clinical Outcomes in Young Athletes: A Controlled, Prospective, Longitudinal Study. Front. Physiol..

[10] Tiernan C., Lyons M., Comyns T., Nevill A.M., Warrington G. (2020). Salivary IgA as a Predictor of Upper Respiratory Tract Infections and Relationship to Training Load in Elite Rugby Union Players. J. Strength Cond. Res..

[11] Lindsay A., Lewis J., Scarrott C., Draper N., Gieseg S.P. (2015). Changes in Acute Biochemical Markers of Inflammatory and Structural Stress in Rugby Union. J. Sports Sci..

[12] Maya J., Marquez P., Peñailillo L., Contreras-Ferrat A., Deldicque L., Zbinden-Foncea H. (2016). Salivary Biomarker Responses to Two Final Matches in Women’s Professional Football. J. Sports Sci. Med..

[13] Moreira A., Bacurau R.F.P., Napimoga M.H., Arruda A.F.S., Freitas C.G., Drago G., Aoki M.S. (2013). Salivary il-21 and iga responses to a competitive match in elite basketball players. Biol. Sport.

[14] Freitas C.G., Aoki M.S., Arruda A.F.S., Franciscon C., Moreira A. (2016). Monitoring Salivary Immunoglobulin A Responses to Official and Simulated Matches In Elite Young Soccer Players. J. Hum. Kinet..

[15] Moreira A., Arsati F., de Oliveira Lima-Arsati Y.B., de Freitas C.G., de Araujo V.C. (2011). Salivary Immunoglobulin a Responses in Professional Top-Level Futsal Players. J. Strength Cond. Res..

[16] Peñailillo L., Maya L., Niño G., Torres H., Zbinden-Foncea H. (2015). Salivary Hormones and IgA in Relation to Physical Performance in Football. J. Sports Sci..

[17] Rico-González M., Pino-Ortega J., Ardigò L.P. (2021). Playing Non-Professional Football in COVID-19 Time: A Narrative Review of Recommendations, Considerations, and Best Practices. Int. J. Environ. Res. Public Health.

[18] Moher D., Liberati A., Tetzlaff J., Altman D.G. (2009). Preferred Reporting Items for Systematic Reviews and Meta-Analyses: The PRISMA Statement. PLoS Med..

[19] O’Reilly M., Caulfield B., Ward T., Johnston W., Doherty C. (2018). Wearable Inertial Sensor Systems for Lower Limb Exercise Detection and Evaluation: A Systematic Review. Sports Med..

[20] Lindsay A., Lewis J., Scarrott C., Gill N., Gieseg S., Draper N. (2015). Assessing the Effectiveness of Selected Biomarkers in the Acute and Cumulative Physiological Stress Response in Professional Rugby Union through Non-Invasive Assessment. Int. J. Sports Med..

[21] Mariscal G., Vera P., Platero J.L., Bodí F., de la Rubia Ortí J.E., Barrios C. (2019). Changes in Different Salivary Biomarkers Related to Physiologic Stress in Elite Handball Players: The Case of Females. Sci. Rep..

[22] Nunes J.A., Crewther B.T., Viveiros L., Jr D.D.R., Aoki M.S. (2011). Effects of Resistance Training Periodization on Performance and Salivary Immune-Endocrine Responses of Elite Female Basketball Players. J. Sports Med. Phys. Fit..

[23] Milanez V.F., Ramos S.P., Okuno N.M., Boullosa D.A. (2014). Evidence of a Non-Linear Dose-Response Relationship between Training Load and Stress Markers in Elite Female Futsal Players. J. Sports Sci. Med..

[24] Caetano Júnior P.C., Carvalho Aguiar J., Ferreira-Strixino J., José Raniero L. (2017). Isokinetic Muscle Performance and Salivary Immune-Endocrine Responses in Handball Players by Fourier Transform Infrared Spectroscopy. Rev. Andal. De Med. Deporte.

[25] Moreira A., de Moura N.R., Coutts A., Costa E.C., Kempton T., Aoki M.S. (2013). Monitoring Internal Training Load and Mucosal Immune Responses in Futsal Athletes. J. Strength Cond. Res..

[26] Moreira A., Arsati F., de Oliveira Lima-Arsati Y.B., Simões A.C., de Araújo V.C. (2011). Monitoring Stress Tolerance and Occurrences of Upper Respiratory Illness in Basketball Players by Means of Psychometric Tools and Salivary Biomarkers. Stress Health.

[27] Nunes J.A., Moreira A., Crewther B.T., Nosaka K., Viveiros L., Aoki M.S. (2014). Monitoring Training Load, Recovery-Stress State, Immune-Endocrine Responses, and Physical Performance in Elite Female Basketball Players During a Periodized Training Program. J. Strength Cond. Res..

[28] Cunniffe B., Griffiths H., Proctor W., Davies B., Baker J.S., Jones K.P. (2011). Mucosal Immunity and Illness Incidence in Elite Rugby Union Players across a Season. Med. Sci. Sports Exerc..

[29] Orysiak J., Witek K., Zembron-Lacny A., Morawin B., Malczewska-Lenczowska J., Sitkowski D. (2017). Mucosal Immunity and Upper Respiratory Tract Infections during a 24-Week Competitive Season in Young Ice Hockey Players. J. Sports Sci..

[30] Coad S., Gray B., Wehbe G., McLellan C. (2015). Physical Demands and Salivary Immunoglobulin A Responses of Elite Australian Rules Football Athletes to Match Play. Int. J. Sports Physiol. Perform..

[31] He C.-S., Tsai M.-L., Ko M.-H., Chang C.-K., Fang S.-H. (2010). Relationships among Salivary Immunoglobulin A, Lactoferrin and Cortisol in Basketball Players during a Basketball Season. Eur. J. Appl. Physiol..

[32] de Arruda A.F.S., de Freitas C.G., de Moura N.R., Aoki M.S., Moreira A. (2013). Resposta imuno-endócrina associada à partida de futsal. Mot. Rev. Educ. Fis..

[33] Nunes J.A., Crewther B.T., Ugrinowitsch C., Tricoli V., Viveiros L., de Rose D., Aoki M.S. (2011). Salivary Hormone and Immune Responses to Three Resistance Exercise Schemes in Elite Female Athletes. J. Strength Cond. Res..

[34] Koch A.J., Wherry A.D., Petersen M.C., Johnson J.C., Stuart M.K., Sexton W.L. (2007). Salivary immunoglobulin aresponse to a collegiate rugby game. J. Strength Cond. Res..

[35] Coad S., Gray B., McLellan C. (2016). Seasonal Analysis of Mucosal Immunological Function and Physical Demands in Professional Australian Rules Footballers. Int. J. Sports Physiol. Perform..

[36] Moreira A., Crewther B., Freitas C.G., Arruda A.F.S., Costa E.C., Aoki M.S. (2012). Session RPE and Salivary Immune-Endocrine Responses to Simulated and Official Basketball Matches in Elite Young Male Athletes. J. Sports Med. Phys. Fit..

[37] Moreira A., Arsati F., Cury P.R., Franciscon C., Simões A.C., de Oliveira P.R., de Araújo V.C. (2008). The Impact of a 17-Day Training Period for an International Championship on Mucosal Immune Parameters in Top-Level Basketball Players and Staff Members. Eur. J. Oral Sci..

[38] Orysiak J., Witek K., Malczewska-Lenczowska J., Zembron-Lacny A., Pokrywka A., Sitkowski D. (2019). Upper Respiratory Tract Infection and Mucosal Immunity in Young Ice Hockey Players During the Pretournament Training Period. J. Strength Cond. Res..

[39] Yamauchi R., Shimizu K., Kimura F., Takemura M., Suzuki K., Akama T., Kono I., Akimoto T. (2011). Virus Activation and Immune Function during Intense Training in Rugby Football Players. Int. J. Sports Med..

[40] Moraes H., Aoki M., Freitas C., Arruda A., Drago G., Moreira A. (2017). SIgA Response and Incidence of Upper Respiratory Tract Infections during Intensified Training in Youth Basketball Players. Biol. Sport.

[41] Mortatti A.L., Moreira A., Aoki M.S., Crewther B.T., Castagna C., de Arruda A.F.S., Filho J.M. (2012). Effect of Competition on Salivary Cortisol, Immunoglobulin A, and Upper Respiratory Tract Infections in Elite Young Soccer Players. J. Strength Cond. Res..

[42] Nehlsen-Cannarella S.L., Nieman D.C., Fagoaga O.R., Kelln W.J., Henson D.A., Shannon M., Davis J.M. (2000). Saliva Immunoglobulins in Elite Women Rowers. Eur. J. Appl. Physiol..

[43] Moreira A., Freitas C.G., Nakamura F.Y., Drago G., Drago M., Aoki M.S. (2013). Effect of Match Importance on Salivary Cortisol and Immunoglobulin A Responses in Elite Young Volleyball Players. J. Strength Cond. Res..

[44] Teeuw W., Bosch J.A., Veerman E.C.I., Nieuw Amerongen A.V. (2004). Neuroendocrine Regulation of Salivary IgA Synthesis and Secretion: Implications for Oral Health. Biol. Chem..

[45] Hucklebridge F., Clow A., Evans P. (1998). The Relationship between Salivary Secretory Immunoglobulin A and Cortisol: Neuroendocrine Response to Awakening and the Diurnal Cycle. Int. J. Psychophysiol..

[46] Papacosta E., Nassis G.P. (2011). Saliva as a Tool for Monitoring Steroid, Peptide and Immune Markers in Sport and Exercise Science. J. Sci. Med. Sport.

[47] Mazanec M., Nedrud J., Kaetzel C., Lamm M. (1993). A Threetiered View of the Role of IgA in Mucosal Defense. Immunol Today.

[48] Gleeson M., Bishop N., Oliveira M., McCauley T., Tauler P., Muhamad A.S. (2012). Respiratory Infection Risk in Athletes: Association with Antigen-Stimulated IL-10 Production and Salivary IgA Secretion: Biomarkers of Infection Risk in Athletes. Scand. J. Med. Sci. Sports.

[49] Bishop N.C. (2009). Acute and Chronic Effects of Exercise on Markers of Mucosal Immunity. Front. Biosci..

[50] Novas A.M., Rowbottom D.G., Jenkins D.G. (2003). Tennis, Incidence of URTI and Salivary IgA. Int. J. Sports Med..

[51] Moreira A., Mortatti A.L., Arruda A.F.S., Freitas C.G., de Arruda M., Aoki M.S. (2014). Salivary IgA Response and Upper Respiratory Tract Infection Symptoms During a 21-Week Competitive Season in Young Soccer Players. J. Strength Cond. Res..

[52] Morgans R., Orme P., Anderson L., Drust B., Morton J.P. (2014). An Intensive Winter Fixture Schedule Induces a Transient Fall in Salivary IgA in English Premier League Soccer Players. Res. Sports Med..

[53] Fahlman M.M., Engels H.-J. (2005). Mucosal IgA and URTI in American College Football Players: A Year Longitudinal Study. Med. Sci. Sports Exerc..

[54] Gleeson M. (2004). Immune Function and Exercise. Eur. J. Sport Sci..

